# Heresy, False Doctrine and Schism

**Published:** 1885-07

**Authors:** 


### HERESY, FALSE DOCTRINE AND SCHISM.

It was to be expected that the new code men, having intrigued so succesfully at Copenhagen, would not consent to be quietly ousted. Some resistance was expected, but, really, we were not prepared to see two powerful and leading medical journals —although known to have new code proclivities, apparently so under the control or influence of that class, as to resort to sophistry, and artful and disingenuous arguments, and a series of special pleadings in their behalf.

The proposition before the medical world is:

Did the instructions under which this committee of arrangements acted, give them authority to organize the congress, and appoint its officers and sections?

The two main points involved in the proposition, incidental thereto, and hinging upon it, are:

1st. In all America, is there nowhere else to be found medical talent of a quality and quantity suitable to take part in a medical congress, except in a few northeastern cities?

2nd. Shall the American Medical Association's Committee appoint as delegates and representatives in a Medical Congress, aliens and enemies—*irregulars*,—in preference to its own members?

Notwithstanding the pronounced and emphatic negative given to the question at New Orleans, by the American Medical Association, the *New York Medical Record* dogmatically asserts the contrary.

Disposing thus of the main question, by *an assertion*, backed by no evidence that we have seen, the *New York Medical Record* and the *New York Medical Journal* then proceed to defend the two absurd propositions by a series of most specious but fallacious arguments; and, loosing temper, they resort to inuendo, and ridicule of the South and West, in a manner at once surprising and unjust.

No *argument* can be made in defense of either proposition that will not do violence at once to reason and common sense; hence these Journals resort to the use of such expressions as that "The South and West have coaxed the American Medical Association into doing a meaningless act."—*Record.*

"The South and West have pulled the Association's nose."—*N. Y. Journal.*

"The Association has shown a wonderful proclivity for having its nose pulled."—*N. Y. Journal.*

"The South and West have expended an immense amount of gas."—*N. Y. Journal.*

"A few ambitious gentlemen want to gain prominence in connection with the Congress," etc. etc.—*N. Y. Med. Record.*

These, and similar arguments (?) are put forward by these columbiads of the journalistic batteries, in defense of that admirable arrangement whereby men who have been kicked out of the association of American Physicians for conduct inconsonant with the fundamental principles of the MAGNA CHARTA which is at once the guide and defense of legitimate medicine, may occupy front seats, and play first fiddle, to the exclusion of their betters, because, forsooth the *Record* says they are "scientific."

But, perhaps, the most ill-natured, unfounded and unjust remark into which our powerful, but misguided, and apparently wilfully blinded cotemporary has allowed its temper to trap it, is, that the gentlemen who were added to the committee, one from each state, are :

☞"Men of whom we know nothing or too much."—*N. Y. Record*, June 20.

In view of the fact that these gentlemen were *picked out* by the State delegations who,in turn,were selected by the StateMedical Associations,it is fair to presume that they are,at least,average representative members of the profession *of the whole Union*, and such a slur as above quoted from the *N. Y. Record*, is an insult to the whole profession of America.

Whatever the *Record* may,or may not know of the representatives of the northern and eastern states—we cannot speak for them—we know enough of those chosen from the southern and western states, to feel assured *our rights* entrusted to their keeping, will be sacred. They will be defended, moreover, with all that chivalry and high sense of honor, which has ever characterized southern and western men, even granting the superior "science" of northern brethren, as demanded by the *Record* ; and the *Record* may be assured that neither accusations, insinuations, nor false reasoning, will avail to persuade them to betray those who trust them ! And such comparisons as that the South, *in its inferiority scientifically*, *daring* to aspire to *any* representation in an International Congress, is like "a youth aping manhood, and making a spectacle," is little calculated to bring about that era of "peace" the *Record* affects to desire.

As illustrating our text further, we refer to "Geography vs. Science," (*N. Y. Record*, June 20) where we are accused of laying claims to southern representation on a geographical basis, and the fact that "a certain state" raises so much cotton. True, we remarked incidently, to illustrate the magnitude of the injustice done Texas in common with other states, and to show her greatness in other relations, that her cotton crop sums up annually, a million and a half bales. True, we did claim that *some* regard should be had to geographical distribution, seeing the profession of America is somewhat homogenious,and meet on terms of social and professional equality; but the *Record* knows we do not base any claim on that alone, and its assertion seems a strange perversion of our meaning. Yet it shows still further that straws will be grasped to prop a lame defense of an action which, we venture, the profession of the world, with the facts clearly before them, will pronounce monstrous.

The *Record* says impressively: "Bear in mind the International Medical Congress has a separate and a higher existence than the American Medical Association." So, indeed has the British Parliament; but it is a *non sequitur* that the new code men are entitled to a seat in either. But, granting that they have as much right in the International Congress as any other men—and nobody denies it—upon what principle of equity, right or justice can they expect to enter it through the portals of the American Medical Association? The idea is absurd, and to ask or expect it, much less demand and insist upon it, is the extreme limit of that quality now universally denominated "cheek." Is it not specifically provided that the International Congress shall be composed of representatives of all respectable organized medical bodies? The New York Academy of Medicine is largely, if not entirely, composed of new code men; they have their own county organizations. Why are not these persons content to go as representatives of their own kind and class? *Suum cuique*! Because they know, not being in affiliation with legitimate medicine, the congress will not admit them, and they wish to sail in, therefore, under cover of the American Medical Association! Well, it won't win!

As a final and strongly put plea against a redistribution of the appointments, the *Record* argues that what the Congress wants is "professors," "authors," teachers, and boasts that the "institutions of learning" are all north! And that ninety-five per cent. of the books are written by northern men! We grant the North can furnish more teachers, professors and authors, but, because of this, are the average physicians North and East more able or learned than their brethren? Or is it conclusive, therefore, that there is *no* learning, *no* science elsewhere? Are the papers read at medical conventions by northern members so vastly superior to those by members from other sections? We are not prepared to admit it. Upon what meat doth these, our medical Cæsars, feed? Was not Sims a southern man? Nott? McDowell? Drake? Gross? Is not Thomas a southern man? Are not many of our physicans graduates of the Philadelphia and New York schools? Granting that ninety-five per cent. of the books *are* written North, we still think our Brother of the *Record* should give the rest of creation credit for the remaining five per cent. of wisdom! These journals, we say, are teaching false doctrine. In their defense ot the appointment of the new code men to represent the American Medical Association they are guilty of heresy; and to keep up the discord by this species of specious, but fallacious reasoning, is daily extending the unfortunate schism that now divides the American medical profession.

The sooner the *Record* counsels its readers to acquiesce in the discision of the American Medical Association with regard to the action of the committee of arrangements, and goes to work to bring about that peace on a just and equitable basis, the sooner we will have an International Congress in America of which the world will not be ashamed. Its present course is keeping up the discord which it affects to deprecate, by singing the syren song of peace, urging "policy," and appealing to the sense of pride, which every true American feels; but, is peace so sweet as to be purchased at the price of principle? "Forbid it, Almighty God."

